# Efficacy and safety of Toliparibumab for the treatment of non-small cell lung cancer: a systematic review and meta-analysis

**DOI:** 10.3389/fonc.2024.1444312

**Published:** 2024-11-07

**Authors:** Yihao Liu, Liangyu Yang, Zhixuan Duan, Qian Cheng, Minghui Liu, HongBing Zhang, HongLin Zhao

**Affiliations:** ^1^ Department of Lung Cancer Surgery, Tianjin Medical University General Hospital, Tianjin, China; ^2^ School of Medicine, Nankai University, Tianjin, China

**Keywords:** Toripalimab, non-small cell lung cancer (NSCLC), efficacy, adverse events, lung cancer, lung tumor

## Abstract

**Purpose:**

This research intends to investigate the treatment of non-small cell lung cancer (NSCLC) using Toripalimab, focusing on its effectiveness and safety profile. Efficacy refers to the survival prognosis, while safety pertains to the occurrence of adverse events in our study. It also aims to provide reference information for neoadjuvant and postoperative therapies.

**Methods:**

Up to March 20, 2024, studies on randomized controlled trials and single-arm trials involving Toripalimab for NSCLC were sourced from the Cochrane Library, Embase, PubMed, and Web of Science databases. Data extraction and analysis were independently conducted by two researchers utilizing Stata 15.0 and R software.

**Results:**

A total of 8 studies were analyzed, including 6 single-arm studies and 2 randomized controlled trials (RCTs). Toripalimab treatment in the RCTs showed an overall survival (OS) of [HR=0.67, 95% CI (0.53, 0.85); p=0.71]. The objective response rate (ORR) from single-arm studies was reported as [ES=0.59, 95% CI (0.36, 0.81); p<0.01], and progression-free survival (PFS) was [ES=4.89, 95% CI (2.65, 9.02); p<0.01]. Furthermore, observed adverse effects included Anemia [OR=0.53, 95% CI (0.26, 0.79); p<0.01], Neutropenia [OR=0.43, 95% CI (0.20, 0.68); p<0.01], and Thrombocytopenia [OR=0.28, 95% CI (0.18, 0.43); p<0.01].

**Conclusions:**

Toripalimab, being China’s first domestically developed anti-tumor PD-1 antibody drug, shows potential advantages over traditional chemotherapy in possibly prolonging patients’ survival times. However, the limited number of studies included indicates the need for additional single-arm and RCT studies to further validate these findings.

**Systematic review registration:**

https://www.crd.york.ac.uk/PROSPERO/, identifier (CRD42024519806).

## 1 Introduction

Globally, lung cancer holds the highest mortality rate among all cancers, with smoking identified as the leading risk factor, followed by air quality concerns (1, 2). Recent data reveal a stabilization and gradual decline in the number of male patients, while the number of female patients has increased significantly. Many of these women are non-smokers, which is largely associated with EGFR mutations (3). Lung cancer is categorized into small cell lung cancer and non-small cell lung cancer (NSCLC), with NSCLC comprising 85% of lung cancer cases. The predominant types of NSCLC are squamous cell carcinoma, large cell carcinoma, and adenocarcinoma. For early-stage NSCLC, the primary treatment method is surgical resection, with about 30% considered resectable (4). Traditional chemotherapy is often the first line of treatment for advanced NSCLC, but its efficacy is limited (5), and it is only suitable for patients without genetic mutations (6). The 5-year overall survival rate is 2 to 13% for NSCLC recurrence and 4% for advanced NSCLC patients (6–8). Among clinical checkpoint inhibitors, anti-programmed death protein 1 (PD-1)/programmed death-ligand 1 (PD-L1) inhibitors are the most effective, as they inhibit downstream pathways and restore T cell antitumor functions (9). Numerous studies indicate that combination therapy can enhance immunotherapy effects by exposing tumor antigens and increasing tumor mutational burden, and preoperative use of immune checkpoint inhibitors (ICIs) can improve survival rates (10). The highest mutation rate in lung adenocarcinoma involves EGFR, reaching up to 20%, with an incidence rate of NSCLC in Asians between 40-60%, making EGFR a critical factor for NSCLC. Resistance is inevitable following the use of TKIs, necessitating combination therapy (11). Current research shows that chemotherapy can induce PD-L1 expression, creating a synergistic effect leading to better survival outcomes (12). Nivolumab combined with platinum-based doublet chemotherapy has received FDA approval for preoperative treatment in resectable NSCLC patients, and atezolizumab is approved for postoperative treatment in patients with PD-L1 > 1%, due to its significant effects and improved clinical outcomes (13–15). Toripalimab, China’s first domestically produced PD-1 antibody (9), is used in melanoma, nasopharyngeal carcinoma, and urothelial carcinoma, with relatively fewer studies in NSCLC. In the treatment of unresectable or metastatic melanoma, Toripalimab was first approved and can provide long-term survival benefits. Additionally, it is approved as a first-line treatment for unresectable locally advanced or recurrent/metastatic esophageal squamous cell carcinoma, ALK non-squamous NSCLC, and metastatic nasopharyngeal carcinoma (12). Therefore, adding PD-1 drugs as a first-line treatment could represent a new breakthrough for most late-stage cancer patients. This meta-analysis focuses on evaluating the effectiveness and safety of Toripalimab in NSCLC patients.

## 2 Methods

This study is registered in the International Prospective Register of Systematic Reviews database (PROSPERO: CRD42024519806).

### 2.1 Search strategy

We searched for articles published up to March 2024 in PubMed, Embase, Cochrane, and Web of Science databases related to the use of Toripalimab in non-small cell lung cancer (NSCLC) patients. General search terms included “non-small cell lung cancer” and “Toripalimab.” The detailed search strategies are provided in **Supplementary Tables S1**-**S4**.

### 2.2 Selection criteria

We included single-arm studies or randomized controlled trials (RCTs) with at least 10 participants. Patients included must be diagnosed with non-small cell lung cancer (NSCLC), primarily consisting of subtypes such as pulmonary adenocarcinoma, squamous cell carcinoma, adenosquamous carcinoma, large cell carcinoma, and sarcomatoid carcinoma, among others. Additionally, patients must receive treatment with Toripalimab either as monotherapy or in combination with standard chemotherapy agents such as pemetrexed, carboplatin, cisplatin, and paclitaxel. The primary endpoints were objective response rate (ORR), progression-free survival (PFS), overall survival (OS), and adverse events (AE). We have excluded literature and cases involving small cell lung cancer or tumors that have metastasized to the lungs from other parts of the body. In terms of interventions, we have also excluded studies where Toripalimab is used in combination with other immunotherapies or targeted drugs. Additionally, We excluded case reports, reviews, conference proceedings, animal studies, meta-analyses, and abstracts. Studies with fewer than 10 participants and those without available full text and data were also excluded. After excluding these articles and removing duplicates, we conducted a meta-analysis.

### 2.3 Data extraction

Two researchers independently performed data extraction. In cases of discrepancies or differing opinions, a third researcher participated in the discussion and decision-making. Extracted data included the author’s name, publication year, basic patient characteristics (gender, average age), tumor staging, ECOG performance status, PD-1 expression levels, intervention measures, and outcome indicators.

### 2.4 Quality assessment

For non-randomized single-arm studies, we used the Methodological Index for Non-Randomized Studies (MINORS), applying the first eight items of this scale, each scored from 0 to 2, for a total maximum score of 16 (16), as shown in **Supplementary Table S5**. For RCTs, we used Version 2 of the Cochrane tool for assessing risk of bias in randomized trials (RoB2) to evaluate the risk of bias, detailed in **Supplementary Figure S1**. The included studies were categorized as having high, moderate, or low risk of bias. Additionally, we define I²<25% as low heterogeneity, 25-75% as moderate heterogeneity, and >75% as high heterogeneity (17).

### 2.5 Statistical analysis

Two researchers independently conducted data extraction and analysis, with any disagreements resolved through discussion with a third researcher. For single-arm studies, effect size (ES) and 95% confidence intervals were used to determine the objective response rate (ORR) and adverse events (AE), while median progression-free survival (PFS) was represented by median PFS. For RCT studies, overall survival (OS) was assessed using hazard ratios (HR) and 95% confidence intervals. Heterogeneity among studies was evaluated using Cochran’s Q test and the I² statistic, with a P-value of less than 0.05 considered statistically significant, indicating substantial differences. Due to variability in participant populations across studies, heterogeneity was expected. Consequently, a random-effects model was employed for the meta-analysis when I² exceeded 50%; otherwise, a fixed-effects model was used. Sensitivity analysis was performed for analyses with significant heterogeneity. Egger’s test was used for linear regression testing, and funnel plots were employed to visually assess publication bias. Analyses were conducted using Stata 17.0 (Stata Corporation, College Station, TX, USA) and R version 3.6.0.

## 3 Results

### 3.1 Literature search

**Figure 1:**
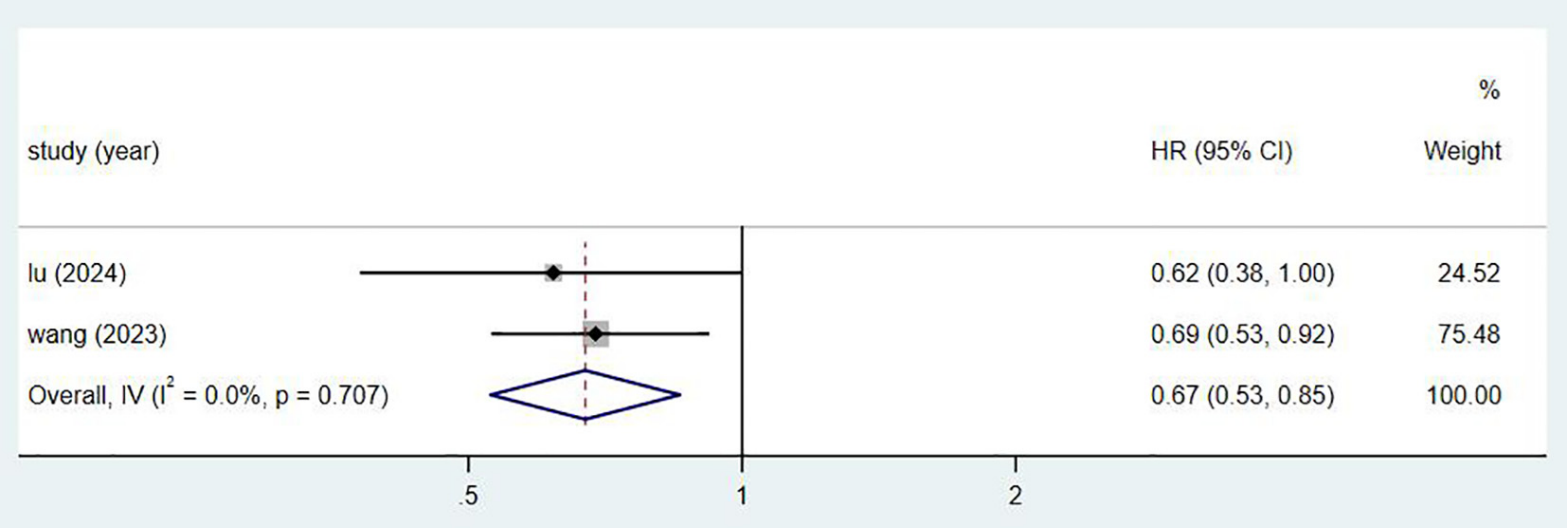
Research search process and results chart.

Initially, we retrieved a total of 743 articles from these databases. We excluded 129 duplicates. After reviewing titles and abstracts, 597 articles were further excluded, including reviews, conference proceedings, case reports, and animal studies. Of the remaining 17 studies, 9 were excluded due to incomplete data. Finally, 8 studies were deemed eligible for inclusion in this meta-analysis. The process of retrieval and screening is illustrated in **Figure 1**.

### 3.2 Study characteristics

Among the 8 included studies, 2 were randomized controlled trials (RCTs) with complete data (8, 18). Both the control and experimental groups in these studies included chemotherapy, using pemetrexed plus platinum for adenocarcinoma and paclitaxel plus platinum for squamous cell carcinoma. The remaining 6 studies were single-arm studies, each with more than 10 participants (19–24). It is hereby noted that the chemotherapy drugs used in all studies may not be identical; however, all of these drugs are standard chemotherapy agents for the treatment of lung cancer. Age, gender, tumor staging, ECOG performance status, PD-1 expression, intervention measures, and outcome measures were recorded in **Table 1**. The study by Wang (21) discussed the efficacy of Toripalimab produced by different processes, which divided the study into two groups. However, due to the small number of subjects included in this study, we combined the two groups to process the data. Since there are only two RCT studies, we extracted part of the data and treated it as single-arm study data. This approach enhances the persuasiveness of the data in this paper.

**Table 1:** Research abstracts included in the meta-analysis.

Single arm											
Study	Year	Sample size	Mean age	Gender(M/F)	Clinical Stage	Pathology	Genetic Mutation	ECOG(0/1)	PD-1(≥1%)	Intervention	Outcome
Jiang	2021	40	47.5	19/21	NR	NR	EGFR mutations	2/38	21	Toripalimab: 240mg/360mg IV once/3week	1. mPFS 2. ORR 3. AE
Tao	2023	55	61.5	50/5	IIB-IIIB	LUAD 9 LUSC 44 ASC 2	NR	NR	NR	Toripalimab: 240mg IV once/3weeks	1.AE
Hou	2023	11	60.7	10/1	II-IIIB	LUAD 2 LUSC 9	KRAS TP53	NR	NR	Toripalimab: 240mg IV once/3weeks	1. ORR 2. AE
Zhao	2021	33	61	27/6	III	LUAD 13 LUSC 18 LELC 3	NR	NR	NR	Toripalimab: 240mg IV once/3weeks	1.AE
Zhu	2022	50	66.0	42/8	IIB-IIIC	LUAD 9 LUSC 32 Others 9	NR	NR	13	Toripalimab: 240mg IV once/3weeks	1. ORR 2. AE
Wang	2020	41	A:59.5 B:57.0	A:15/5 B;14/7	III-IV	LUAD 29 LUSC 10 Others 2	NR	A:7/13 B:6/15	NR	T:Toripalimab:(200/500L) 3mg/kg IV once/2weeks	1. Mpfs 2. ORR 3. AE
RCT											
Study	Year	Sample size	Mean age	Gende (M/F)	Clinical Stage	Pathology	Clinical Stage	ECOG(0/1)	PD-1(≥1%)	Intervention	Outcome
Wang	2023	T:309 C:156	T:63 C:61	377/88	IIIB-IVB	LUSC 220 Non-LUSC 245	NR	T:66/243 C:36/120	T:201(+)/108(-) C:103/53	T:Toripalimab: IV 240mg, once/3week C:Placebo: IV 240mg,once/3week	1. ORR 2. mPFS 3. OS 4. AE
Lu	2024	T:202 C:202	T:62 C:61	T:181/21 C:189/13	II-III	LUSC 90 Non-LUSC 314	NR	T:70/132 C:73/129	T:133/51/18 C:132/54/16	T:Toripalimab: IV 240mg, once/3week C:Placebo: IV 240mg, once/3week	1. ORR 2. OS 3. AE

T, treatment group; C control group; mPFS, median progression free survival; ORR, objective response rate; AE, adverse event; OS, overall survival; IV, intravenous injection; LUAD, lung adenocarcinoma; LUSC, lung squamous cell carcinoma; LELC, Lymphoepithelioma-like carcinoma; NR, not reported.

### 3.3 Study data for the single-arm studies

#### 3.3.1 ORR

**Figure 2:**
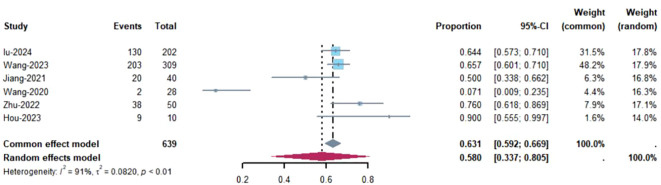
Estimated proportion of ORR in Toripalimab-treated patients for single-arm studies.

Objective response rate (ORR) refers to the proportion of tumor patients who achieve complete response (CR) or partial response (PR) after treatment, primarily assessed by imaging to evaluate changes in tumor size. Pathological complete response (pCR) refers to the absence of residual viable cancer cells in the resected surgical specimen after neoadjuvant therapy, as determined by pathological examination (25). Of the 8 studies, 6 reported the Objective Response Rate (ORR) (8, 18, 19, 21, 23, 24). These six studies included a total of 639 patients, with an I² value of 91% and P<0.01, indicating significant heterogeneity. Therefore, a random-effects model was applied, as shown in **Figure 2**. The figure demonstrates that the ORR for NSCLC patients treated with Toripalimab in combination with chemotherapy was [ES=0.58, CI(0.6-0.81), P=0.00]. A funnel plot, shown in **Supplementary Figure S2**, visually suggests the presence of publication bias (results of Egger and Begg tests). A sensitivity analysis conducted in **Supplementary Figure S3** indicated that removing the study by Wang (21) significantly decreased the heterogeneity (I²=57%, P<0.01), suggesting that this study could be a major source of the observed heterogeneity. We believe that the main cause of heterogeneity is the low ORR in this study, coupled with the small sample size. However, this result is also within our expectations.

#### 3.3.2 PFS

**Figure 3:**
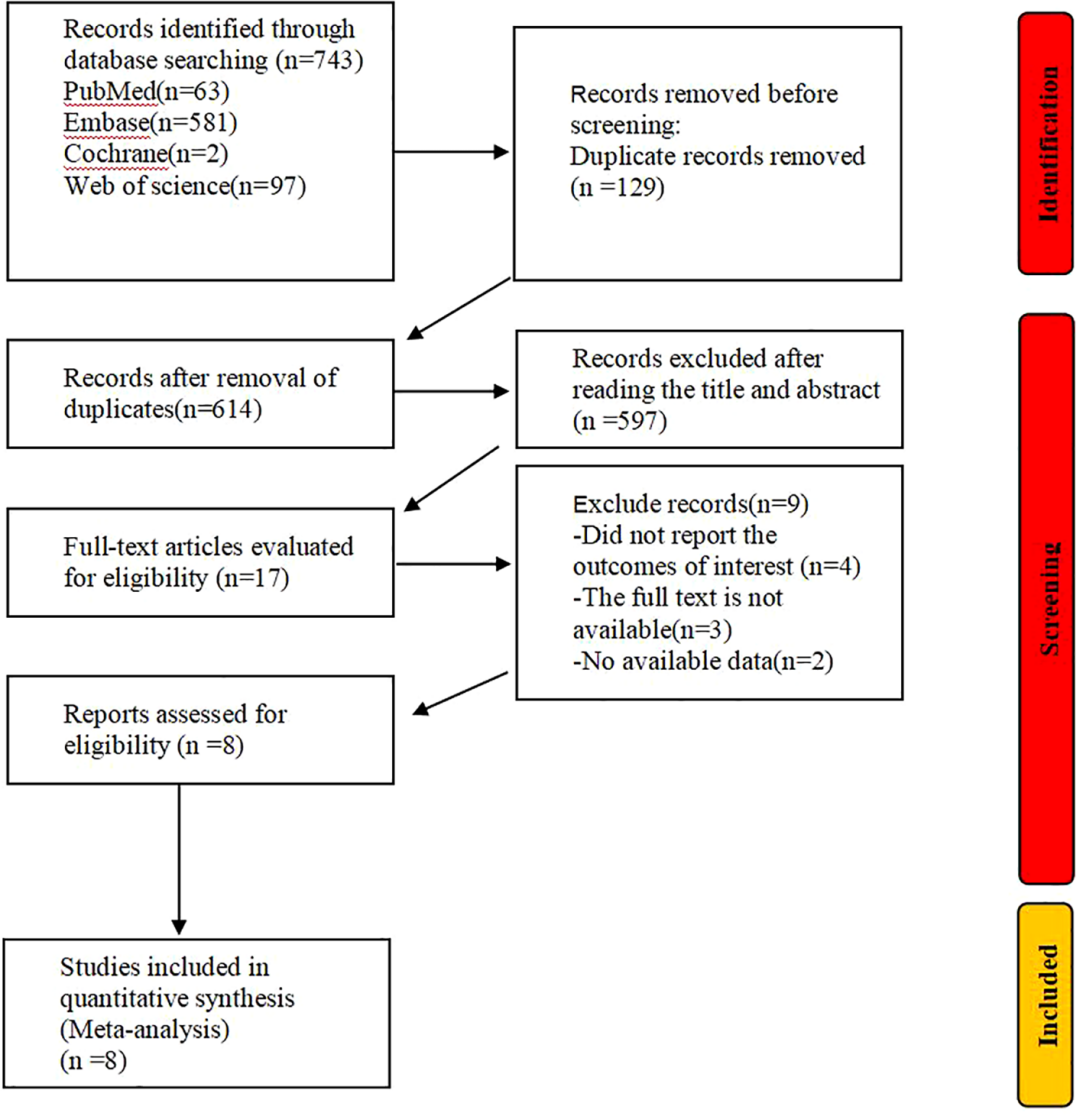
Estimated proportion of mPFS in Toripalimab-treated patients for single-arm studies.

Three studies reported the median Progression-Free Survival (mPFS) (18, 21, 23), involving a total of 546 patients, with an I² value of 96.3% and P<0.00. **Figure 3** shows that the mPFS for NSCLC patients treated with Toripalimab in combination with chemotherapy was [median PFS = 5.49 months, 95% CI (2.88, 10.49); P=0.00]. A sensitivity analysis, shown in **Supplementary Figure S4**, proved to be relatively stable. The possibility of publication bias appears to be low, as indicated by the Egger test (0.469) and Begg test (0.469).

#### 3.3.3 Meta-analysis for adverse events

In this study, in addition to efficacy, we also investigated the safety by examining the types of adverse events or the occurrence of more severe conditions. Below are the results of the adverse events. The 8 studies reported adverse event (AE) information (8, 18–24), involving a total of 740 patients. The main adverse events included anemia, neutropenia, leukopenia, thrombocytopenia, nausea, rash, thyroid dysfunction, ALT increase, and AST increase. The effect sizes (ES) for any grade of AEs were: anemia=64%, neutropenia=50%, leukopenia=45%, thrombocytopenia=30%, nausea=27%, rash=19%, thyroid dysfunction=20%, hemoptysis=10%, ALT increase=31%, and AST increase=31%. For severe adverse events (Grade ≥3), the ES were: anemia=11%, neutropenia=28%, leukopenia=19%, thrombocytopenia=7%, decreased appetite=1%, pneumonia=4%, and rash=1%. These data are visually presented in **Table 2**.

**Table 2:** Adverse events of toliparibumab for NSCLC therapy.

adverse event	Any grade				Grade≥3			
	study	Heterogeneity		ES (95%CI)	study	Heterogeneity		ES (95%CI)
		P	I^2^(%)			P	I^2^(%)	
Anemia	5	<0.01	96	0.64[0.44,0.81]	5	<0.01	90	0.11[0.05,0.22]
Neutropenia	6	<0.01	98	0.50[0.27,0.73]	6	<0.01	98	0.28[0.12,0.45]
Leukopenia	5	<0.01	98	0.45[0.11,0.83]	5	<0.01	99	0.19[0.00,0.56]
Thrombocytopenia	7	<0.01	95	0.30[0.20,0.46]	6	<0.01	86	0.07[0.02,0.13]
Decreased appetite	3	0.23	31	0.32[0.28,0.36]	3	0.51	0	0.01[0.00,0.02]
Nausea	6	<0.01	90	0.27[0.16,0.38]	6	0.97	0	0.001[0.00,0.007]
Vomiting	3	<0.01	90	0.13[0.02,0.24]	3	0.98	0	0.000[0.000,0.007]
Pneumonia	3	<0.01	81	0.07[0.01,0.37]	3	<0.01	85	0.04[0.00,0.14]
Fatigue	5	<0.01	96	0.20[0.08,0.32]	5	0.92	0	0.004[0.000,0.012]
Constipation	3	0.52	0	0.20[0.17,0.24]	None			
Rash	7	0.10	43	0.19[0.16,0.22]	6	0.72	0	0.01[0.00,0.02]
Thyroid dysfunction	7	<0.01	92	0.20[0.11,0.28]	6	0.98	0	0.000[0.000,0.003]
Hemoptysis	3	0.24	31	0.10[0.08,0.12]	3	0.22	33	0.01[0.00,0.02]
Diarrhea	3	0.05	68	0.06[0.02,0.23]	3	0.52	0	0.013[0.000,0.026]
Alt increase	5	<0.01	91	0.31[0.16,0.45]	5	0.62	0	0.009[0.001,0.021]
Ast increase	5	<0.01	80	0.31[0.18,0.45]	5	0.23	29	0.006[0.001,0.014]

CI, confidence interval; NR, Not reported.

### 3.4 Meta-analysis for RCT

#### 3.4.1 OS

**Figure 4:**
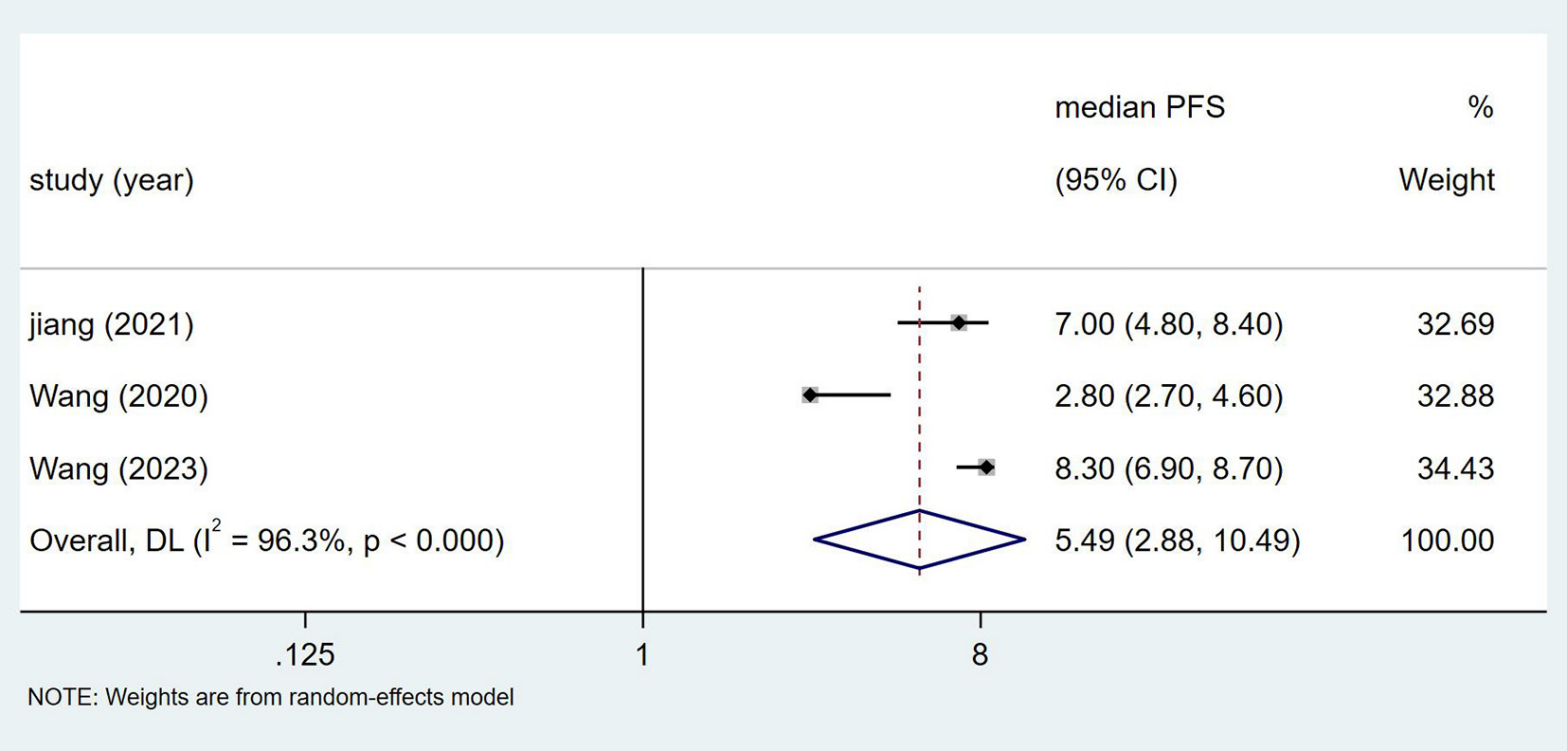
Estimated proportion of OS in Toripalimab-treated patients for RCT studies.

Both randomized controlled trials (RCTs) reported on overall survival (OS) (8, 18), involving a total of 869 patients. **Figure 4** shows that the effect size (hazard ratio, HR) for OS is 0.69, with a confidence interval (CI) of (0.53, 0.85).

## 4 Discussion

According to our investigation, this is the inaugural study on Toripalimab in the context of non-small cell lung cancer (NSCLC), with the latest literature emerging in 2024 by Lu et al. (8). The novelty of this research lies in examining domestically developed immunotherapy agents for NSCLC, demonstrating their efficacy while revealing that the adverse effects of this medication remain within an acceptable limit. In first-line treatment for NSCLC lacking driver gene mutations, conventional therapies primarily include chemotherapy. Nevertheless, some research indicates that the anti-tumor immune response triggered by chemotherapy following tumor cell death could augment the effectiveness of tumor immunotherapy (26). The FDA has granted approval for Toripalimab to treat advanced nasopharyngeal carcinoma. According to Luo’s meta-analysis (27), the median progression-free survival (PFS) for patients treated with Toripalimab alongside gemcitabine and cisplatin is 10.8 months, with a duration of response (DOR) averaging 9.9 months and an overall response rate (ORR) of 88.1%. In contrast, patients with non-squamous NSCLC undergoing treatment with alternative immunotherapy drugs combined with chemotherapy achieved a median PFS of only 8.5 months and a median overall survival (OS) of 17.2 months (26, 28, 29). This underscores the significant promise of this drug or treatment regimen in oncological therapy, suggesting that combining chemotherapy with immunotherapy may yield improved patient outcomes.

For single-arm studies, the ORR was reported as [ES=0.58, CI (0.34, 0.81)], while the mPFS was [ES=5.49, CI (2.9, 10.5)]. The evaluation of randomized controlled trials (RCTs) indicated an OS of [HR=0.67, 95% CI (0.53, 0.85)]. When compared to standard chemotherapy (30), the combination of Toripalimab and chemotherapy showed greater survival advantages. It is important to acknowledge the effectiveness of Toripalimab, but we should treat this research data with caution given the limited number of studies, most of which are single-arm trials. Regarding the ORR, our findings suggest that the variability is largely due to Wang’s study (21), potentially linked to its smaller sample size. In two RCTs, Wang’s research indicated a median PFS of 8.3 months, which is significantly higher than the 5.6 months survival rate observed in the placebo cohort. Furthermore, although Lu’s study did not specify mPFS, it noted that 48.5% of patients receiving Toripalimab exhibited a major pathological response following surgical resection, highlighting Toripalimab’s promising efficacy in treating non-small cell lung cancer. In China, Toripalimab is the first approved PD-1 inhibitor, primarily utilized for nasopharyngeal carcinoma, melanoma, and urothelial carcinoma (9). It is sanctioned as a first-line treatment for adults with metastatic or recurrent locally advanced nasopharyngeal carcinoma, administered in combination with cisplatin and gemcitabine chemotherapy (31). In the study NCT03513666 (23), forty patients were treated with Toripalimab alongside either carboplatin or pemetrexed. The treatment regimen, given every three weeks, resulted in a median OS of 23.5 months, a median PFS of 7.0 months, and an ORR of 50%, substantially enhancing patient survival.

Previous research has highlighted the pathological response as a critical endpoint, which can be categorized into major pathological response (MPR) and complete pathological response (pCR) (29). MPR has been identified as a significant predictor of long-term overall survival (OS) in patients with NSCLC, functioning as a predictive marker. The application of neoadjuvant immunotherapy is on the rise, with both MPR and pCR gaining importance in randomized controlled trial (RCT) studies (29). Neoadjuvant therapies, including immunotherapy or chemotherapy, have demonstrated safety and efficacy for stage I-III NSCLC, particularly in the context of combination neoadjuvant chemo-immunotherapy (32). In the majority of NSCLC cases that proceeded to surgical resection following neoadjuvant chemotherapy, the pathological response acted as an endpoint for treatment, effectively managing tumor micro-metastases and enhancing mediastinal lymph node clearance rates (33). According to Zhao’s study (20), 50% of patients achieved pCR, while two-thirds (20 out of 30) attained MPR. Although the sample size is not sufficiently large to validate Toripalimab as a primary or secondary therapy, it lays the groundwork for future neoadjuvant strategies. The recent research by Lu et al. (8) is particularly noteworthy, encompassing 501 patients, with 404 receiving Toripalimab treatment. Participants underwent three cycles preoperatively and one cycle postoperatively, facilitating an extended treatment duration. The hazard ratio (HR) was recorded at 0.66. In comparison to the placebo group (MPR=0.01), the treatment group yielded a value of 0.24, reflecting an intergroup difference of 23.7% [95% CI, 17.6%-29.8%], P <.001. The article also indicated that the pathological response might serve as a potential surrogate marker for event-free survival. Nonetheless, in our country, there is a tendency toward non-invasive treatments, making its application in preoperative neoadjuvant therapy promising, while post-chemotherapy biopsies continue to present challenges. Chemotherapy enhances immunotherapy by inducing tumor cell apoptosis and releasing antigens that stimulate the immune response. Common side effects associated with standard chemotherapy include nausea, vomiting, hair loss, and suppression of bone marrow function. The risk of experiencing these adverse effects tends to rise, particularly when multiple chemotherapy drugs are used concurrently, resulting in an increased number of complications (34). In contrast, the rate of adverse reactions linked to immunotherapy is notably lower than that associated with chemotherapy, with the majority being gastrointestinal, dermatological, and systemic issues such as fatigue and fever. These reactions can be effectively managed with symptomatic treatments (35). Additionally, chemotherapy enhances the infiltration of cytotoxic T cells into the tumor microenvironment and stimulates inflammatory signaling, which supports the recruitment and activation of immune cells (24). It also reduces the cell burden that immune cells must handle and lowers the levels of immunosuppressive substances produced by tumor cells. Moreover, since immunotherapy alone is often insufficient for tumor eradication, its combination with chemotherapy becomes essential (36). Adverse events related to Toripalimab mainly involve anemia, neutropenia, leukopenia, and reduced appetite, with grade 3 neutropenia occurring in 28% of cases, while other events are less frequent. Overall, the incidence of adverse effects is considered acceptable, and pre-medication can mitigate or eliminate these reactions. Research indicates that the rates of treatment-related adverse events (TRAE) vary from 54% to 76%, with fatal adverse events occurring at a rate between 0.36% and 0.63% (9). Adverse events can be classified into five categories, with the most prevalent reactions—such as nausea, vomiting, decreased appetite, hypothyroidism, rash, fever, diarrhea, constipation, peripheral neuropathy, musculoskeletal pain, cough, upper respiratory infections, insomnia, fatigue, dizziness, and discomfort—occurring at rates of 20% or higher, all within acceptable severity limits. Firstly, the existing literature is limited, particularly regarding studies on Toripalimab for non-small cell lung cancer, especially randomized controlled trials (RCTs). Secondly, non-small cell lung cancer encompasses both squamous cell carcinoma and adenocarcinoma, yet the literature fails to clearly differentiate between them, hindering subgroup analysis. Furthermore, the baseline chemotherapy regimens for squamous cell and adenocarcinomas differ in combined chemotherapy, potentially resulting in significant heterogeneity. Some studies also overlook factors such as smoking history, genetic mutations, family medical history, and prior treatment history, further contributing to variability. Lastly, comparing the incidence of adverse events between immunotherapy and traditional chemotherapy lacks persuasiveness due to differing baselines and a scarcity of studies.

## 5 Conclusion

In summary, this meta-analysis compiled the latest study along with previous literature, and we found that Toripalimab is beneficial for patient survival. It provides some support for the research on Toripalimab for non-small cell lung cancer and also offers new insights into treatment plans for advanced patients with PD-L1 > 1%.

## Data Availability

The original contributions presented in the study are included in the article/**Supplementary Material**. Further inquiries can be directed to the corresponding author.
