# Supplementary material for: Efficacy and safety of Toliparibumab for the treatment of non-small cell lung cancer: a systematic review and meta-analysis
Source: Front Oncol. 2024 Nov 7;14:1444312. doi: 10.3389/fonc.2024.1444312 (PMC11578821; doi:10.3389/fonc.2024.1444312)
Supplement: Supplementary file 1 [file DataSheet1.docx]

| Search Steps | Query |
| --- | --- |
| 1 | "toripalimab"[Supplementary Concept] |
| 2 | "toripalimab"[Title/Abstract] |
| 3 | "toripalimab"[Supplementary Concept] OR "toripalimab"[Title/Abstract] |
| 4 | "Lung Neoplasms"[MeSH Terms] |
| 5 | "lung neoplasms"[Title/Abstract] OR "pulmonary neoplasms"[Title/Abstract] OR "neoplasms lung"[Title/Abstract] OR "lung neoplasm"[Title/Abstract] OR "neoplasm lung"[Title/Abstract] OR "neoplasms pulmonary"[Title/Abstract] OR "neoplasm pulmonary"[Title/Abstract] OR "pulmonary neoplasm"[Title/Abstract] OR "lung cancer"[Title/Abstract] OR "cancer lung"[Title/Abstract] OR "cancers lung"[Title/Abstract] OR "lung cancers"[Title/Abstract] OR "pulmonary cancer"[Title/Abstract] OR "cancer pulmonary"[Title/Abstract] OR "cancers pulmonary"[Title/Abstract] OR "pulmonary cancers"[Title/Abstract] OR "cancer of the lung"[Title/Abstract] OR "cancer of lung"[Title/Abstract] |
| 6 | "Lung Neoplasms"[Title/Abstract] OR "pulmonary neoplasms"[Title/Abstract] OR "neoplasms lung"[Title/Abstract] OR "lung neoplasm"[Title/Abstract] OR "neoplasm lung"[Title/Abstract] OR "neoplasms pulmonary"[Title/Abstract] OR "neoplasm pulmonary"[Title/Abstract] OR "pulmonary neoplasm"[Title/Abstract] OR "lung cancer"[Title/Abstract] OR "cancer lung"[Title/Abstract] OR "cancers lung"[Title/Abstract] OR "lung cancers"[Title/Abstract] OR "pulmonary cancer"[Title/Abstract] OR "cancer pulmonary"[Title/Abstract] OR "cancers pulmonary"[Title/Abstract] OR "pulmonary cancers"[Title/Abstract] OR "cancer of the lung"[Title/Abstract] OR "cancer of lung"[Title/Abstract] OR "Lung Neoplasms"[MeSH Terms] |
| 7 | ("Lung Neoplasms"[Title/Abstract] OR "pulmonary neoplasms"[Title/Abstract] OR "neoplasms lung"[Title/Abstract] OR "lung neoplasm"[Title/Abstract] OR "neoplasm lung"[Title/Abstract] OR "neoplasms pulmonary"[Title/Abstract] OR "neoplasm pulmonary"[Title/Abstract] OR "pulmonary neoplasm"[Title/Abstract] OR "lung cancer"[Title/Abstract] OR "cancer lung"[Title/Abstract] OR "cancers lung"[Title/Abstract] OR "lung cancers"[Title/Abstract] OR "pulmonary cancer"[Title/Abstract] OR "cancer pulmonary"[Title/Abstract] OR "cancers pulmonary"[Title/Abstract] OR "pulmonary cancers"[Title/Abstract] OR "cancer of the lung"[Title/Abstract] OR "cancer of lung"[Title/Abstract] OR "Lung Neoplasms"[MeSH Terms]) AND ("toripalimab"[Supplementary Concept] OR "toripalimab"[Title/Abstract]) |

Supplementary Table S1: PubMed Retrieval strategy

| Search Steps | Query |
| --- | --- |
| 1 | TS=(Lung Neoplasms) OR TS=(Pulmonary Neoplasms) OR TS=(Neoplasms, Lung) OR TS=(Lung Neoplasm) OR TS=(Neoplasm, Lung) OR TS=(Neoplasms, Pulmonary) OR TS=(Neoplasm, Pulmonary) OR TS=(Pulmonary Neoplasm) OR TS=(Lung Cancer) OR TS=(Cancer, Lung) OR TS=(Cancers, Lung) OR TS=(Lung Cancers) OR TS=(Pulmonary Cancer) OR TS=(Cancer, Pulmonary) OR TS=(Cancers, Pulmonary) OR TS=(Pulmonary Cancers) OR TS=(Cancer of the Lung) OR TS=(Cancer of Lung) |
| 2 | TS=(toripalimab) |
| 3 | #2 AND #1 |

Supplementary Table S2: Web of Science Retrieval strategy

| No. | Query |
| --- | --- |
| #24 | #3 AND #23 |
| #23 | #4 OR #5 OR #6 OR #7 OR #8 OR #9 OR #10 OR #11 OR #12 OR #13 OR #14 OR #15 OR #16 OR #17 OR #18 OR #19 OR #20 OR #21 OR #22 |
| #22 | 'cancer of lung':ab,ti |
| #21 | 'cancer of the lung':ab,ti |
| #20 | 'pulmonary cancers':ab,ti |
| #19 | 'cancers, pulmonary':ab,ti |
| #18 | 'cancer, pulmonary':ab,ti |
| #17 | 'pulmonary cancer':ab,ti |
| #16 | 'lung cancers':ab,ti |
| #15 | 'cancers, lung':ab,ti |
| #14 | 'cancer, lung':ab,ti |
| #13 | 'lung cancer':ab,ti |
| #12 | 'pulmonary neoplasm':ab,ti |
| #11 | 'neoplasm, pulmonary':ab,ti |
| #10 | 'neoplasms, pulmonary':ab,ti |
| #9 | 'neoplasm, lung':ab,ti |
| #8 | 'lung neoplasm':ab,ti |
| #7 | 'neoplasms, lung':ab,ti |
| #6 | 'pulmonary neoplasms':ab,ti |
| #5 | 'lung neoplasms':ab,ti |
| #4 | 'lung tumor'/exp |
| #3 | #1 OR #2 |
| #2 | 'toripalimab':ab,ti |
| #1 | 'toripalimab'/exp |

Supplementary Table S3: Embase Retrieval strategy

| ID |  |  |  |  |  |  |  |  |  |  |  |  |  |  |
| --- | --- | --- | --- | --- | --- | --- | --- | --- | --- | --- | --- | --- | --- | --- |
| #1 | MeSH descriptor: [Lung Neoplasms] explode all trees | | | | | | | | | | | | | |
| #2 | (Lung Neoplasms):ti,ab,kw OR (Pulmonary Neoplasms):ti,ab,kw OR (Neoplasms, Lung):ti,ab,kw OR (Lung Neoplasm):ti,ab,kw OR (Neoplasm, Lung):ti,ab,kw | | | | | | | | | | | | | |
| #3 | (Neoplasms, Pulmonary):ti,ab,kw AND (Neoplasm, Pulmonary):ti,ab,kw AND (Pulmonary Neoplasm):ti,ab,kw AND (Lung Cancer):ti,ab,kw AND (Cancer, Lung):ti,ab,kw | | | | | | | | | | | | | |
| #4 | (Cancers, Lung):ti,ab,kw AND (Lung Cancers):ti,ab,kw AND (Pulmonary Cancer):ti,ab,kw AND (Cancer, Pulmonary):ti,ab,kw AND (Cancers, Pulmonary):ti,ab,kw | | | | | | | | | | | | | |
| #5 | (Pulmonary Cancers):ti,ab,kw AND (Cancer of the Lung):ti,ab,kw AND (Cancer of Lung):ti,ab,kw | | | | | | | | | | | | | |
| #6 | #1 or #2 or #3 or #4 or #5 | | | | | | | | | | | | | |
| #7 | (toripalimab):ti,ab,kw | | | | | | | | | | | | | |
| #8 | #4 and #5 | | | | | | | | | | | | | |

Supplementary Table S4: Cochrane Retrieval strategy

| study | year | A clearly stated aim | Inclusion of consecutive patients | Prospective collection of data | Endpoints appropriate to the aim of the study | Unbiased assessment of the study endpoint | Follow-up period appropriate to the aim of the study | Loss to follow up less than 5% | Prospective calculation of the study size |
| --- | --- | --- | --- | --- | --- | --- | --- | --- | --- |
| Jiang | 2 | 2 | 2 | 2 | 2 | 0 | 2 | 2 | 0 |
| Wang | 2 | 2 | 2 | 2 | 2 | 0 | 2 | 2 | 1 |
| Zhu | 2 | 2 | 2 | 2 | 2 | 0 | 2 | 2 | 1 |
| Zhao | 2 | 2 | 2 | 2 | 2 | 0 | 2 | 2 | 0 |
| Hou | 2 | 2 | 2 | 2 | 2 | 0 | 2 | 2 | 0 |
| Tao | 2 | 2 | 2 | 2 | 2 | 0 | 2 | 2 | 0 |

Supplementary Table S5: Quality assessment of single-arm studies on the MINORS scale


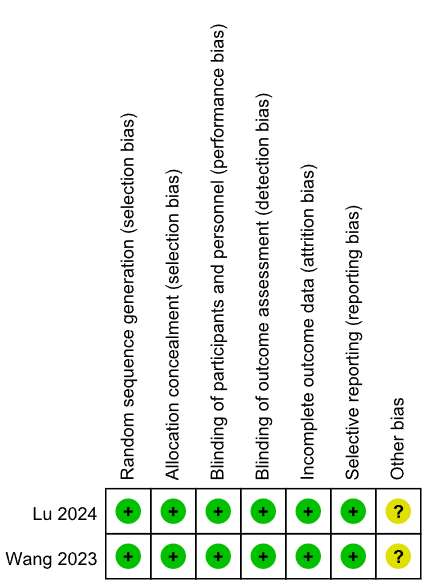

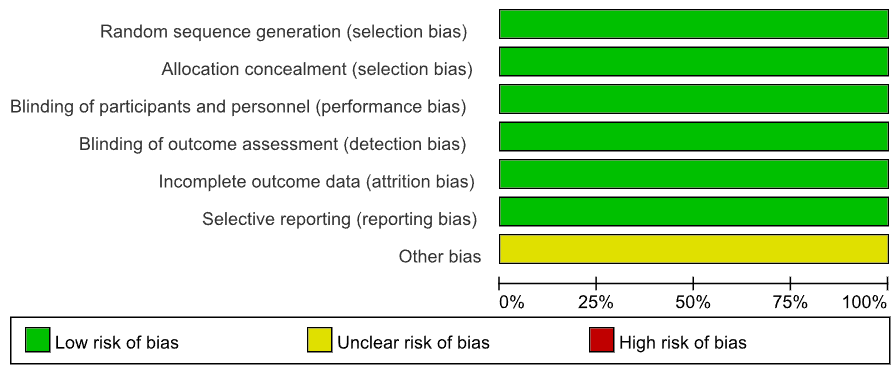


Supplementary Figure S1: Risk of bias assessment. (A) Risk of bias summary; (B) .Risk of bias graph


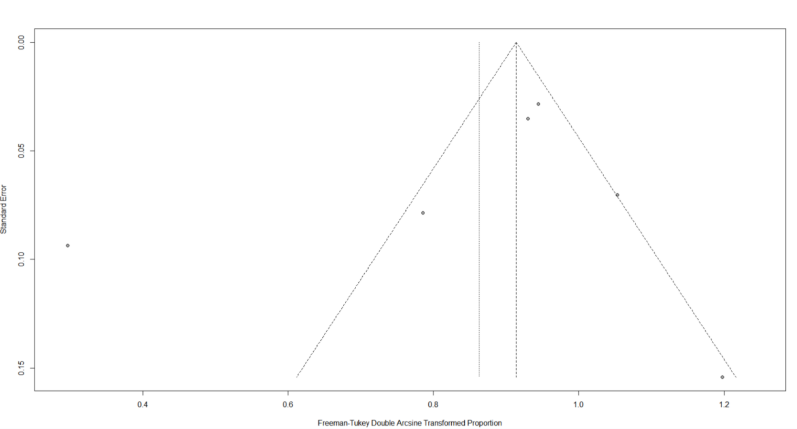


Supplementary Figure S2: Funnel plot of ORR in single arm study


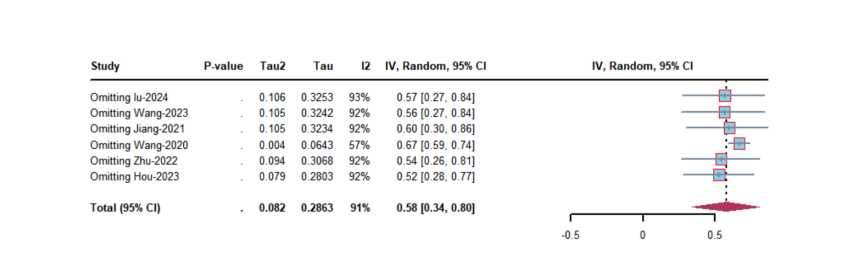


Supplementary Figure S3：Sensitivity analysis of ORR in single arm study


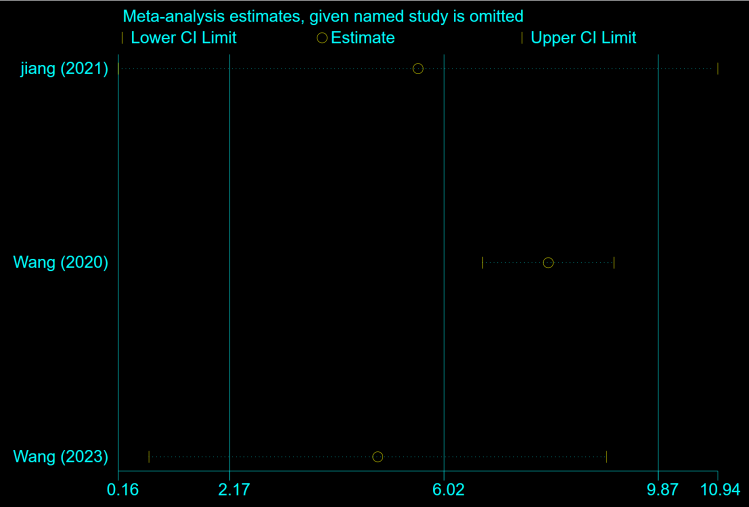


Supplementary Figure S4：Sensitivity analysis of mPFS in single arm study
